# First pregnancy events and future breast density: modification by age at first pregnancy and specific VEGF and IGF1R gene variants

**DOI:** 10.1007/s10552-014-0386-2

**Published:** 2014-05-07

**Authors:** Lee Ann Prebil, Rochelle R. Ereman, Mark J. Powell, Farid Jamshidian, Karla Kerlikowske, John A. Shepherd, Marc S. Hurlbert, Christopher C. Benz

**Affiliations:** 1 Marin County Department of Health and Human Services, 899 Northgate Drive, Suite 415, San Rafael, CA 94903 USA; 2 Departments of Medicine and Epidemiology and Biostatistics, University of California, San Francisco, CA USA; 3 Department of Radiology and Biomedical Imaging, University of California, San Francisco, CA USA; 4 Avon Foundation for Women, New York, NY USA; 5 Buck Institute for Research on Aging, Novato, CA USA; 6 Helen Diller Family Comprehensive Cancer Center, University of California, San Francisco, CA USA

**Keywords:** Breast density, Breast cancer, Pregnancy, Pregnancy-induced hypertension, SNPs, Reproductive history

## Abstract

**Purpose:**

Pregnancy characteristics have been associated with breast cancer risk, but information is limited on their relationship with breast density. Our objective was to examine the relationship between first pregnancy characteristics and later life breast density, and whether the association is modified by genotype.

**Methods:**

The Marin Women’s Study was initiated to examine breast cancer in a high-incidence mammography population (Marin County, CA). Reproductive characteristics and pregnancy information including pregnancy-induced hypertension (PIH) were self-reported at the time of mammography. Forty-seven candidate single nucleotide polymorphisms were obtained from saliva samples; seven were assessed in relation to PIH and percent fibroglandular volume (%FGV). Breast density assessed as %FGV was measured on full-field digital mammograms by the San Francisco Mammography Registry.

**Results:**

A multivariable regression model including 2,440 parous women showed that PIH during first pregnancy was associated with a statistically significant decrease in %FGV (*b* = −0.31, 95 % CI −0.52, −0.11), while each month of breast-feeding after first birth was associated with a statistically significant increase in %FGV (*b* = 0.01, 95 % CI 0.003, 0.02). PIH and breast-feeding associations with %FGV were modified by age at first birth. In a subsample of 1,240 women, there was evidence of modification in the association between PIH and %FGV by specific vascular endothelial growth factor (VEGF) (rs3025039) and insulin growth factor receptor-1 (IGFR1) (rs2016347) gene variants.

**Conclusion:**

These findings suggest that first pregnancy characteristics may exert an influence on extent of breast density later in life and that this influence may vary depending on inherited IGFR1 and VEGF genotypes.

**Electronic supplementary material:**

The online version of this article (doi:10.1007/s10552-014-0386-2) contains supplementary material, which is available to authorized users.

## Background

Parity and age at first birth consistently have been shown to be associated with breast cancer risk in women worldwide (e.g., [1]). The mechanisms underlying the protective effect of pregnancy include a reduction in the number of mammary stem cells, an alteration in the responsiveness of the breast to estrogens, differentiation of mammary epithelial cells, and a change in the levels of circulating hormones (as reviewed in [2]). In addition to the difference in breast cancer risk between nulliparous and parous women, there is evidence that the characteristics of pregnancy, particularly first birth, affect breast cancer risk. An increased risk of breast cancer has been associated with having had a pregnancy that resulted in a preterm birth [3], higher birthweight (e.g., [4]), or multifetal gestation [3], and a decreased risk has been found with having had a pregnancy in which the woman developed hypertension or preeclampsia [3, 5], had a smaller placental weight [6], or had nausea or vomiting [7, 8]. Investigators have found that pregnancy characteristics may be particularly important in the first birth, and with a late age at first birth (e.g., [3]).

The relationship between pregnancy characteristics and breast density has received little attention. Breast density has an established, strong relationship to breast cancer. Research has suggested that this relationship is causal rather than correlational (e.g., [9]) thus providing a potentially important surrogate target for prevention studies. While research has consistently shown a negative association between increasing parity and breast density (e.g., [10, 11]), findings have been less consistent for an association between breast density and older age at first birth (as reviewed in [12]). A few studies have demonstrated that some of the same birth characteristics shown to be associated with increased breast cancer risk were associated with high breast density, specifically, preterm birth, nulliparity/low parity, older age at first birth, and high birth weight [10, 11, 13, 14]. Lope et al. [14] found that duration of breastfeeding was positively associated with breast density, while Butler et al. [10] found no significant association between breast-feeding and breast density.

A better understanding of the association between first pregnancy and breast density could inform breast cancer prevention efforts. If first birth characteristics are found to be associated with breast density in the same direction as their association with breast cancer, this would indicate that these pregnancy events affect breast cancer through their impact on breast density. This study therefore aimed to examine the association between first pregnancy and breast density (Study 1). We additionally endeavored to further explore whether the association between first pregnancy and breast density varied by candidate germline gene variants, specifically single nucleotide polymorphisms (SNPs) known to be related to breast cancer, breast density, or pregnancy characteristics (Study 2).

## Materials and methods

### Study participants

This study was conducted using data from women enrolled in the Marin Women’s Study (MWS). The MWS is a cross-sectional study conducted at mammography centers in Marin County, California associated with Kaiser Permanente, Sutter Health (Novato Community Hospital), and Marin General Hospital. It is estimated that 80 % of mammograms conducted in Marin residents are conducted at centers affiliated with these health care facilities, representing approximately 38,000 women annually. These mammography sites are also included in the San Francisco Mammography Registry (SFMR), one of seven registries included in the National Cancer Institute Breast Cancer Surveillance Consortium. Risk factors and saliva specimens collected in the MWS are linked with breast density, measured as percent fibroglandular volume (%FGV), and breast cancer outcome.

At the time of analysis, there were 11,361 women enrolled in the MWS; for purposes of this analysis (Study 1), women were excluded if they were missing data on age (*n* = 80), if they reported ever having been diagnosed with breast cancer (*n* = 751), if they reported taking antiestrogens (*n* = 107), if they had never had a live birth (*n* = 2,814), if they did not have a measure of breast density assessed as %FGV (*n* = 4,326), if they had missing data on any of the model variables (*n* = 639), if they reported having had a hysterectomy with ovarectomy (*n* = 160), or if their first birth was not a singleton birth (*n* = 44), leaving an analysis population of 2,440.

### Exposure assessment

Primary data collection in the MWS includes self-report information from a questionnaire. Women are asked to report in-depth information on their reproductive history, including information on life course socioeconomic status, alcohol use, and use of exogenous hormones, and data on other established breast cancer risk factors, including family history. Reproductive history includes parity and age at first birth, and for each pregnancy women are asked to report multifetal gestation, duration of breast-feeding, low and high birth weight (<2,500 g, >4,000 g), preterm birth, pregnancy weight gain (pounds), and high blood pressure during pregnancy [or pregnancy-induced hypertension (PIH)] (yes/no). Self-reported height and weight are collected from the SFMR.

At the time of entry into the MWS, women are asked whether they are willing to donate a saliva specimen. Those who consent to donate a saliva sample (89 % of population) are sent a kit in the mail and asked to return the tube of donated saliva in a preaddressed and postage-paid envelope to the Buck Institute for Research on Aging where the saliva is logged, stored, and processed. The specimens are processed by separation into supernatant available for steroid hormone analysis, and a cellular component from which DNA is isolated using Invitrogen’s PureLink Genomic DNA kits. Samples are stored in the MWS Biorepository housed at the Buck Institute in Novato, California.

### Selection of SNP candidates

For Study 2, participants were also selected from the MWS population and were drawn from among 2,400 participants for whom SNP data were available from donated saliva samples.

Twenty-five preselected candidate SNPs were multiplexed and analyzed in the MWS DNA samples by InterGenetics, Inc (Oklahoma City, OK) in parallel with analysis of their panel of 22 OncoVue SNPs. All SNP assays were performed and evaluated as previously described [15], and the CLIA-approved 22 SNP OncoVue assay and algorithm were previously validated as a new individualized breast cancer risk estimator using buccal DNA samples from an earlier (1997–1999) Marin County case–control study cohort [16]. Selection of the novel 25 SNP candidates evaluated in the present study followed a detailed literature search of SNPs reported in GWAS studies to be significantly associated (in at least one published report) to specific exposures, outcomes, and hypothesized breast cancer associated pathways of relevance to the current MWS. In cases where there were more than one SNP from a given gene, weight of literature, linkage characteristics, and potential functionality based on specific location of the SNP within the gene were considered. Gene frequency was also a major selection criterion to ensure adequate power for analysis. For purposes of the analysis examining interactions with PIH reported in this paper, we evaluated SNPs in the following seven genes: eNOS (NOS3), ESR2, VEGF, EDN1, IL-10, HCFXI (KLKB1), IGFR1.

### Outcome assessment

Each participant in the study was required to have undergone a screening mammogram. One of the novel features of this study is the measure of %FGV assessed by the method single-energy X-ray absorptiometry (SXA) [17–20]. This method uses a calibration phantom of the same thickness as the compressed breast, circumventing some of the problems associated with other breast density measures including their subjectivity and lack of absolute reference standards [19]. The first generation calibration phantom (Gamma) used for this method has provided preliminary data on 8,600 women to show that %FGV is reproducible to approximately 2 % between successive measures and accurate to known standards of breast composition using reference phantoms [17]. Investigators have demonstrated that %FGV is inversely correlated with age, body mass index (BMI), and menopausal status, as expected for a measurement of breast density, and is positively associated with breast cancer risk [20]. %FGV data are collected by the SFMR at all sites in Marin County which employ digital mammography and are obtained from the SFMR through a cooperative agreement. Data used in this study were obtained using version 6.5 of the SXA software.

The MWS was approved by the participating institutions, and participants provided written informed consent for participation in the study.

### Statistical analysis

Multivariable linear regression analyses were conducted to examine the associations between %FGV and reproductive factors, controlling for relevant confounders. Robust regression techniques were used to minimize the effects of outliers and influential observations (i.e., observations that have a large impact on the regression analysis). All models controlled for a base set of confounding variables, including current age, BMI, race, education, smoking, family history of breast cancer (whether a first degree relative has been diagnosed with breast cancer), hysterectomy status, menopausal status, and exogenous hormone use at the time of the mammogram. Prior to regression, %FGV was square root transformed to normalize the distribution.

All genotyping data were checked for compliance of single-gene allelic frequencies with Hardy–Weinberg frequency expectations using *χ* ^2^ goodness-of-fit test. Bivariate associations between the genotypes of the SNPs and %FGV were examined. Multivariable linear regression models were constructed adjusting for potential confounders including current BMI, age, race, and age of first live birth, and interaction terms were generated for the genotype of each SNP compared to a baseline genotype. Statistical tests were conducted to examine the interaction term for each level of the SNP compared to the baseline level of the SNP, as well as for the joint interaction effect of each SNP overall and PIH, and *p* values examined for statistical significance.

## Results

The distribution of covariates and the mean %FGV for each level of the covariate are presented in Table 1. The vast majority of the women were White, had a college degree, and were postmenopausal. The mean %FGV was 35.3. In these unadjusted analyses, there were significant associations between %FGV and age, menopausal status, BMI, race, education, smoking status, hysterectomy status, and age at menarche; all associations were in the expected directions.

**Table 1:** Mean percent fibroglandular volume (%FGV) by study population characteristics (Study 1)

Characteristic	Parous women (*n* = 2,440)	
	*n*	Mean %FGV (SD)
All women	2,440	35.3 (21.0)
Age		
≤ 45	481	49.4 (22.5)
46–55	756	39.1 (22.00)
56–65	680	28.4 (16.0)
>65	523	25.8 (14.2)
*F* statistic, *p* value		169.71, *p* < 0.001
Menopausal status		
Premenopausal	940	46.3 (22.7)
Postmenopausal	1,500	28.4 (16.4)
*F* statistic, *p* value		509.2, *p* < 0.001
Current exogenous hormone use		
No	2,042	35.4 (21.4)
Yes	398	34.9 (18.8)
*F* statistic, *p* value		0.18, *p* = 0.67
Body mass index (BMI)		
Underweight (<18.5)	56	62.6 (25.9)
Normal weight (18.5–24.9)	1,525	42.3 (20.5)
Overweight (25–29.9)	578	23.2 (11.6)
Obese (30 +)	260	16.4 (9.0)
*F* statistic, *p* value		305.45, *p* < 0.001
Race		
White	2,214	35.1 (21.00)
Black	9	32.3 (25.3)
Asian	96	40.4 (21.6)
Other	41	41.9 (23.2)
Hispanic	80	31.6 (16.5)
*F* statistic, *p* value		3.13, *p* = 0.01
Education		
HS or less	109	28.1 (17.1)
Some college	614	29.7 (17.1)
College or post graduate	1,717	37.8 (21.9)
*F* statistic, *p* value		41.80, *p* < 0.001
Smoking History		
Never	1,383	37.7 (21.9)
Current	74	32.00 (18.1)
Former	983	32.1 (19.3)
*F* statistic, *p* value		21.81, *p* < 0.001
Hysterectomy status		
No	2,192	36.3 (21.2)
Yes	248	26.8 (17.1)
*F* statistic, *p* value		46.41, *p* < 0.001
Menarche		
10 or younger	93	29.8 (19.3)
11–14	2,089	34.7 (20.6)
15+	258	41.8 (22.8)
*F* statistic, *p* value		16.72, *p* < 0.001

The distribution of reproductive characteristics and the mean %FGV for the different levels of the characteristics is presented in Table 2. In this population, 47.9 % gave birth for the first time at age 30 or older and 23 % had only one birth. There were significant crude associations between %FGV and months of breast-feeding, parity, age at first birth, birthweight, and PIH.

**Table 2:** Mean percent fibroglandular volume (%FGV) by first pregnancy risk factors (Study 1)

Characteristic	Parous women (*n* = 2,440)	
	*n*	Mean %FGV (SD)
Months of breast-feeding		
0	460	27.2 (15.4)
1–3	434	32.4 (18.7)
4–6	547	35.2 (20.9)
7–12	666	39.1 (21.9)
>12	333	42.9 (24.1)
*F*, *p*		37.72, *p* < 0.001
Parity		
1	563	36.8 (22.5)
2	1,219	36.1 (21.0)
3	482	34.0 (19.7)
4	142	29.0 (17.5)
5+	34	25.6 (16.5)
*F*, *p*		6.66, p < 0.001
Age at first birth		
<20	110	25.9 (17.0)
20–29	1,161	30.8 (18.1)
30–34	680	39.5 (21.7)
35+	489	42.2 (23.6)
*F*, *p*		55.37, *p* < 0.001
Birthweight		
Low	178	33.1 (20.2)
High	262	32.1 (19.5)
Normal	2,000	35.9 (21.2)
*F*, *p*		4.84, *p* = 0.01
Pregnancy high blood pressure		
No	2,284	35.6 (21.0)
Yes	156	30.9 (19.8)
*F*, *p*		7.21, *p* = 0.01
Weeks gestation		
38+	2,227	35.3 (21.1)
36–37	156	36.4 (20.7)
≤35	57	32.00 (18.3)
*F*, *p*		0.92, *p* = 0.40
Pregnancy weight gain (lbs)		
0 or weight loss	5	20.8 (13.9)
1–10	50	35.0 (21.3)
11–25	912	35.2 (21.1)
26–40	1,064	35.3 (20.7)
>40	409	35.8 (21.5)
*F*, *p*		0.65, *p* = 0.62

Table 3 presents the results of multivariable models examining the effect of first birth characteristics on breast density overall and stratified by age at first birth. Because the results are presented in units corresponding to the square root transformation of %FGV, the point estimates are not interpretable on a %FGV scale, but can be interpreted in terms of the magnitude of the effect.

**Table 3:** Linear regression model: percent fibroglandular volume (%FGV) in parous MWS women with %FGV measurement and model variables (Study 1)

	Overall (*n* = 2,440)	Age at first birth	
		<30 (*n* = 1,271)	30 + (*n* = 1,169)
Parity (number: 1–5)	0.02 (−0.04, 0.08)	0.01 (−0.06, 0.08)	−0.05 (−0.16, 0.07)
High blood pressure (vs. no)	−0.31 (−0.52, −0.11)*	−0.24 (−0.53, 0.06)	−0.42 (−0.71, −0.13)*
Breast-feeding (months)	0.01 (0.003, 0.02)*	0.01 (0.0002, 0.03)^†^	0.01 (0.001, 0.02)*
Gestational weight gain (lbs)	−0.002 (−0.01, 0.002)	−0.0001 (−0.01, 0.01)	−0.002 (−0.01, 0.01)
Birthweight (vs. normal)			
Low	−0.04 (−0.26, 0.18)	−0.29 (−0.57, 0.002)^†^	0.05 (−0.31, 0.40)
High	−0.04 (−0.21, 0.12)	0.01 (−0.21, 0.22)	−0.02 (−0.28, 0.23)
Weeks gestation (versus 38 + weeks)			
36–37 weeks	0.08 (−0.14, 0.29)	0.24 (−0.05, 0.54)	0.03 (−0.28, 0.35)
<35 weeks	−0.02 (−0.40, 0.35)	0.23 (−0.29, 0.76)	−0.002 (−0.55, 0.54)
Menarche (vs. <10)			
11–14	0.07 (−0.19, 0.33)	0.07 (−0.24, 0.39)	−0.08 (−0.52, 0.37)
15+	0.26 (−0.04, 0.56)^†^	0.32 (−0.05, 0.69)^†^	−0.04 (−0.54, 0.47)
Age at first birth (vs. <20)			
20–29	−0.01 (−0.26, 0.24)	NA	NA
30–34	0.01 (−0.26, 0.28)		
35+	0.13 (−0.15, 0.41)		
*R* ^2^	0.49	0.38	0.44

Controlled for current age, BMI, race, education, smoking, first degree relative with breast cancer, hysterectomy status, menopausal status (except in models stratified by hysterectomy status), and hormone use at the time of the mammogram

* Differences significant at the *p* < 0.05 level

^†^Differences significant at the *p* < 0.10 level

Overall, having experienced high blood pressure during the first pregnancy was associated with a significantly lower %FGV (−0.31; 95 % CI −0.52, −0.11). In addition, each month of breast-feeding was associated with a significantly increased %FGV (0.01; 95 % CI 0.003, 0.02). Late menarche (age 15+ vs. before age 10) was associated with a borderline significant increase in %FGV (0.26; 95 % CI −0.04, 0.56). This model explained 49 % of the variance in %FGV.

Models were stratified by whether the first birth occurred before age 30 or at age 30 or later to examine whether these associations varied by age at first birth (Table 3). Among women with a first birth before age 30, each month of breast-feeding was borderline significantly associated with an increase in breast density (0.01; 95 % CI 0.0002, 0.03). In addition, having had a low birthweight infant was associated with a borderline significantly decrease in %FGV (−0.29; 95 % CI −0.57, 0.002), and having had a late menarche was associated with a borderline significant increase in %FGV (0.32; 95 % CI −0.05, 0.69). In women whose first birth occurred at age 30 or later, the model estimates were similar to those in the overall population. Having experienced high blood pressure during the first pregnancy was associated with a significant decrease in %FGV (−0.42; 95 % CI −0.71, −0.13), and months of breast-feeding were associated with a significant increase in %FGV (0.01; 95 % CI 0.001, 0.02). While the model explained 44 % of the variance in %FGV in women whose first birth was age 30 or greater, it explained 38 % of the variability in women whose first birth was age <30 years.

Because of the significant protective associations between PIH and %FGV, and the consistency of this finding with those in the literature indicating a significantly protective effect of PIH on breast cancer, we analyzed salivary DNA samples to determine whether interactions existed for the associations between PIH and %FGV based on seven SNPs previously shown to be associated with PIH (Online Resource 1). For this analysis, participants were excluded if they had a history of breast cancer or use of antiestrogens, if they did not have a valid %FGV measurement, or if they had never had a live birth, leaving an analysis sample size of 1,240, which was further reduced in multivariable models because of missing data on one or more of the variables included in the model, including SNP results when genotyping was not successful for that SNP. In line with the Study 1 analysis that was limited to reproductive characteristics in the first pregnancy, our Study 2 definition for the exposure was limited to the development of PIH in first pregnancy. All of the seven SNPs were found to be in compliance with Hardy–Weinberg frequency expectations. In this Study 2 subset, %FGV was lower in women with a history of PIH, although this finding did not reach statistical significance. None of the bivariate associations between the genotypes of the seven selected SNPs and %FGV were statistically significant.

**Fig. 1:**
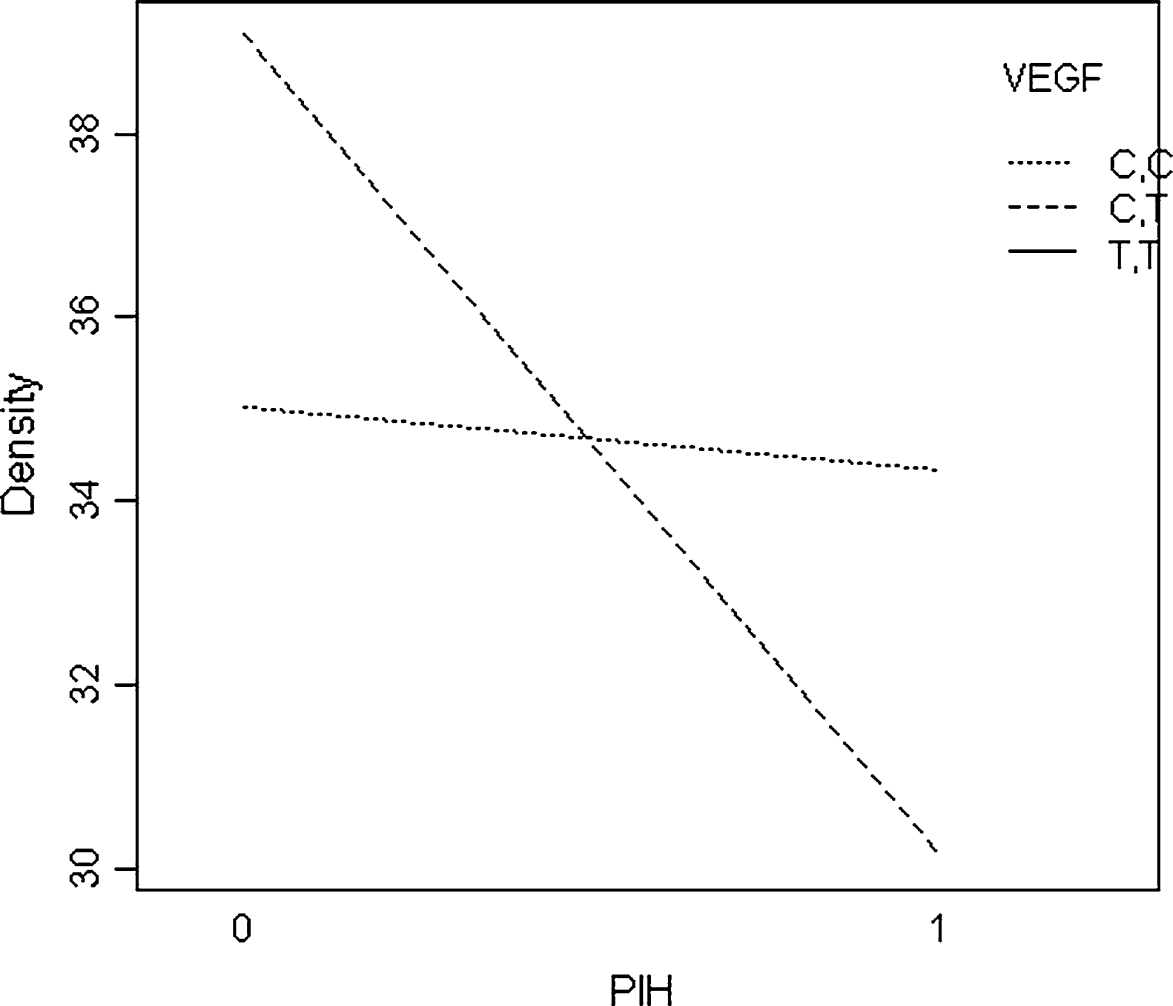
Interaction of PIH and VEGF

**Fig. 2:**
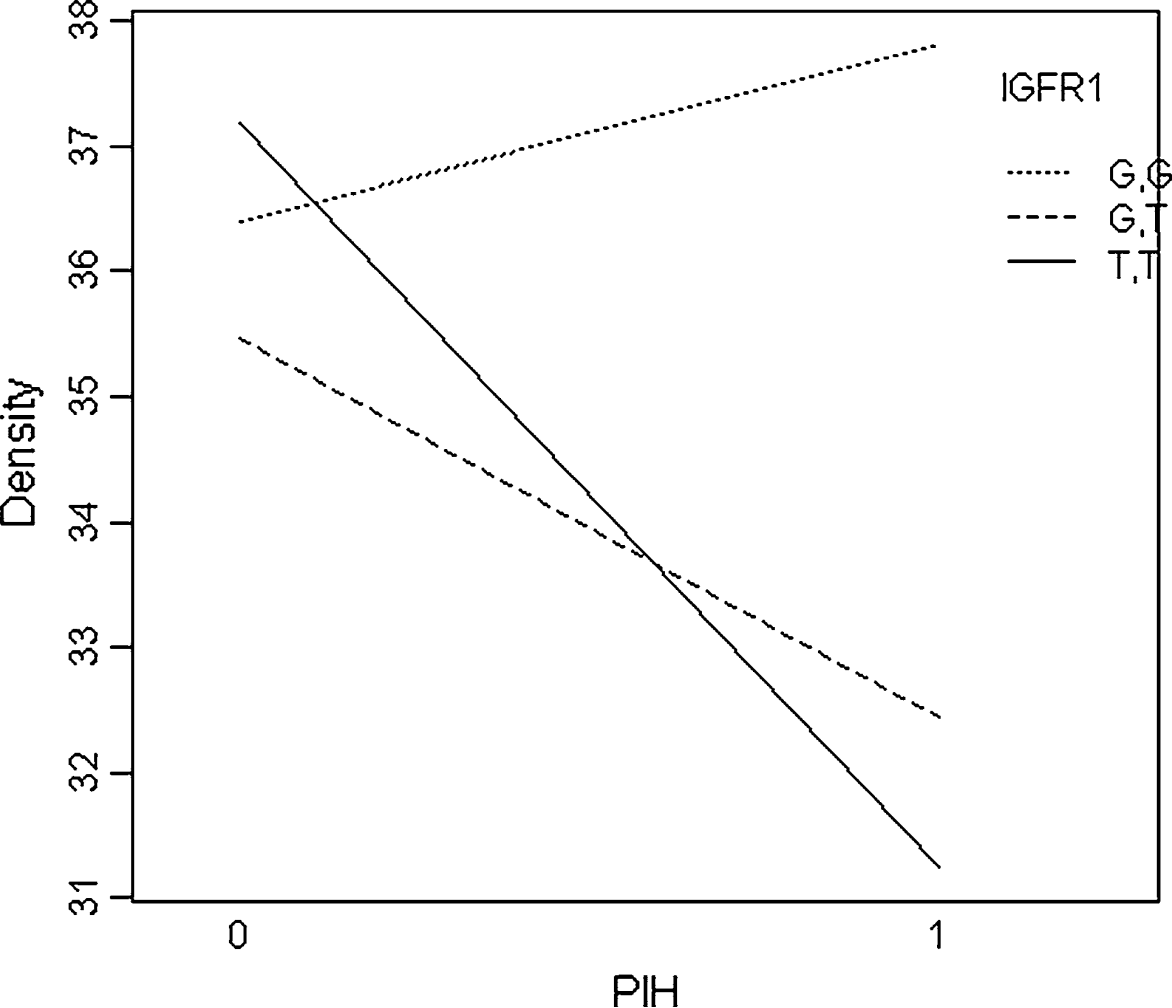
Interaction of PIH and IGFR1

Multivariable analyses indicated no significant interactions between PIH and five of the seven SNPs tested (Table 4); however, two of the seven SNPs were associated with PIH. A borderline significant interaction was found between the CT genotype of the VEGF SNP and PIH on %FGV (*p* = 0.063) (compared to the CC genotype). The TT genotype, which has the lowest frequency, did not occur in any of the women with PIH in the first pregnancy and thus does not appear in the results. Statistically significant interactions were found between PIH and the GT genotype of the IGFR1 SNP (*p* = 0.01) (compared to the GG genotype); those with PIH and the GT genotype had significantly lower %FGV than those with GG genotype of the IGFR1 SNP (joint interaction term *p* value = 0.03). A borderline significant interaction was found between the TT genotype of the IGFR1 SNP and %FGV (compared to the GG genotype) (*p* = 0.07). This group was only half the size of the GT group, which may explain why, despite the apparent larger effect seen in the graphic, this did not reach statistical significance. The main effects terms for the GT and TT genotypes of the IGFR1 SNP were both 0.18 in this multivariable model that included the interaction terms. Both of these sets of interactions are presented visually in Figs. 1 and 2, below.

**Table 4:** Multivariate associations between genotypes of the 7 PIH SNPs and percent fibroglandular volume (%FGV) (Study 2)

SNP	Baseline genotype	Interaction term	Estimate	*p* value	Joint test *p* values for interaction	*n* For each genotype
EDN	GG					471
		EDN_GT×PIH	−0.178	0.58		266
		EDN_TT×PIH	0.300	0.70	0.77	38
HCFX	CC					374
		HCFX_CT×PIH	−0.004	0.99		323
		HCFZ_TT×PIH	−0.263	0.57	0.84	75
NOS3	CC					124
		NOS3_CT×PIH	0.075	0.85		342
		NOS3_TT×PIH	0.176	0.68	0.91	305
IL10	CC					186
		IL10_CT×PIH	−0.020	0.95		365
		IL10_TT×PIH	−0.116	0.76	0.95	214
VEGF	CC	VEGF_CT×PIH	−0.621	0.06	NA	557
						200
IGFR1	GG					174
		IGFR1_GT×PIH	−0.906	0.01		389
		IGFR1_TT×PIH	−0.734	0.07	0.03	195
ESR2	CC					131
		ESR2_CT×PIH	0.403	0.31		379
		ESR2_TT×PIH	−0.053	0.90	0.60	251

In this case, it can be seen that having PIH and the CC genotype resulted in no change in %FGV, while having PIH with the CT genotype resulted in a decrease in %FGV.

## Discussion

In this cross-sectional study, we found that PIH during first pregnancy was associated with significantly reduced breast density measured by %FGV in later life, the effect of which was greatest in those women whose first birth occurred at age 30 or later, where preeclampsia and gestational hypertension (or PIH) are more prevalent. To our knowledge, this is the first study to identify an association between experience of a pregnancy affected by PIH/gestational hypertension and %FGV. PIH has been fairly consistently associated with a reduction in breast cancer risk (e.g., [5, 21]), though some studies have found no (e.g., [6, 22]) or even increased risk [23, 24]. PIH is a multifactorial disease with genetic and environmental factors known to be involved in its etiology although full understanding of its pathogenesis remains elusive despite decades of research [25]. Likewise, the mechanism by which PIH affects breast cancer risk is unknown although potentially via the same etiologic basis as PIH, with theories focusing on placental dysfunction and a subsequent lowering of circulating estrogens [26], increase in serum insulin-like growth factor [27], and/or angiogenic factors [28]. In support of the theory of an altered hormonal milieu are findings such as those of Cerhan et al. [29] who found non-significantly reduced breast density in women born from a pregnancy in which their mothers experienced eclampsia/preeclampsia [29], and similar findings reported in a systematic review by Xue and Michels [30] who found significantly decreased risk of breast cancer if a woman’s mother had experienced preeclampsia or eclampsia.

In this study, PIH was shown to significantly interact with specific allelic variants of insulin growth factor receptor-1 (IGFR1; rs2016347), and borderline significantly with a specific allelic variant of vascular endothelial growth factor-A (VEGF-A; rs3025039) genes, indicating that the protective effects of first pregnancy PIH on later life breast density and breast cancer risk may depend on inherited functional variants in these two growth factor receptor and angiogenic factor genes. Prior studies involving these allelic variants support this conclusion, since the VEGF-A SNP investigated (rs3025039) has been repeatedly linked to decreased plasma levels of VEGF and reduced breast cancer risk, [31–33] while the IGFR1 SNP investigated (rs2016347) has not only been previously associated with breast density but also found to be an independent prognostic marker for breast cancer recurrence [34, 35]). While these various sources of evidence make it unlikely that these two SNPs found to modulate the significant association between PIH and %FGV were simply false positive discoveries, these novel observations require additional validation in larger population-based studies given the strong inheritance pattern underlying breast density.

Duration of breast-feeding in first pregnancy was associated with increased %FGV later in life in our overall study population and in women regardless of age at first birth (though these findings were of borderline significance). Our finding of a positive association between duration of breast-feeding and %FGV is in agreement with that of Lope et al. [14], who found a positive association between breast density (using the cumulus method) and duration of lactation, but conflicts with those of others who found no association (e.g., [10, 25]) or an inverse association between breast-feeding and breast density (e.g., [36]). Differences in the timing and measurement of breast density between these diverse studies are the most likely explanation for their disparate associations, though this could also be due to basic differences in the study populations. Although breast-feeding has been thought to produce an overall protective effect against breast cancer (e.g., [37]), many studies find no association (e.g., [22]) and recent systematic reviews of the topic fail to support an association (e.g., [38, 39]). More research is needed in the area of breast-feeding and breast health to better elucidate whether a protective effect exists, and if so, when and how breast-feeding might affect breast density.

A number of other first pregnancy factors were less consistently associated with %FGV, but were suggestive in certain sub-analyses, including gestational weight gain, infant birthweight, and preterm birth. Other authors have found associations between breast density and birthweight [11, 14] and preterm birth and breast density [11]. While our data were suggestive of relationships between %FGV and these birth characteristics, our findings were neither consistent nor strong. The differences may be due to differences in the breast density measure or characteristics of the study populations, including differences in the age structure (only 55 and younger in El-Bastawissi et al. [11] and 45–68 in Lope et al. [14]).

This study has a number of important strengths, including a large sample size, a contemporary sample, a novel measure of breast density, and the availability of a wide variety of reproductive characteristics for study. The primary limitation in this study is the use of self-reported data for reproductive history. Though it is possible that women may not accurately recall information about their first pregnancy, particularly if it occurred in the distant past, we would expect that they would accurately recall the major events including their age, an experience of PIH, and breast-feeding. To the extent that recall bias is present, it would be nondifferential (i.e., not associated with %FGV), and would thus bias the results toward the null. Another limitation is that, despite the fact that the overall sample size in this study was large, the sample size was small for specific subgroup analyses. Studies with larger populations may be better able to detect significant associations between birth characteristics and breast density where they exist. Selection bias may be present in the sample of patients providing saliva samples for the SNP analyses; women who consented to donate saliva were significantly more likely to be of White Non-Hispanic race and to be of higher socioeconomic status based on education and income, but were not significantly different in terms of family history of breast cancer or current age. While this may limit the generalizability of the findings indicating an interaction between PIH and VEGF and IGFR1 on %FGV, any selection bias present should not limit the validity of the findings. Finally, this study was intentionally restricted to first births, but it will be important to determine whether the findings for first birth characteristics hold for all births or whether they are unique to the first birth (e.g., whether total duration of breast-feeding has the same association with breast density as duration of breast-feeding after the first birth).

## Conclusion

In summary, we found associations between first birth characteristics and breast density measured as %FGV that confirmed and extended the few published findings on birth characteristics and breast density. PIH was associated with a decrease in breast density and breast-feeding a increase in breast density, which may help elucidate the pathway by which they operate to affect breast cancer. Variation in the association between PIH and %FGV by genotype of IGFR1 and VEGF suggest that the protective effect of PIH on breast density may vary between women depending on genotype.

## Electronic supplementary material

Below is the link to the electronic supplementary material.

Supplementary material 1 (DOCX 12 kb)

## Abbreviations

%FGV: Percent fibroglandular volume
MWS: Marin Women’s Study
SNPs: Single nucleotide polymorphisms
PIH: Pregnancy-induced hypertension
SFMR: San Francisco Mammography Registry
SXA: Single X-ray absorptiometry
IGFR1: Insulin growth factor receptor-1
VEGF-A: Vascular endothelial growth factor-A
