# Supplementary material for: First pregnancy events and future breast density: modification by age at first pregnancy and specific VEGF and IGF1R gene variants
Source: Cancer Causes Control. 2014 May 7;25(7):859–68. doi: 10.1007/s10552-014-0386-2 (PMC4048469; doi:10.1007/s10552-014-0386-2)
Supplement: Supplementary file 1 — Supplementary material 1 (DOCX 12 kb) [file 10552_2014_386_MOESM1_ESM.docx]

**Online Resource 1. SNPs examined for interaction on association between PIH and Percent Fibroglandular Volume (%FGV)**

| **Gene** | **Gene name** | **SNP ID** | **Base change** | **Allele Frequency** | **Gene Functions** |
| --- | --- | --- | --- | --- | --- |
| *eNOS*  *(NOS3)* | Nitric oxide  synthase 3 | rs2070744***3-*** | T→C | 0.29, 0.71 | Anti-tumor activities/ Vascular tone |
| *ESR2* | Estrogen receptor 2 (ER beta) | rs928554  ***ESR2-*** | A→G | 0.64, 0.36 | Hormone Receptors/ Steroid Hormone Metabolism |
| *VEGF* | Vascular endothelial growth factor A | rs3025039  ***VEGF-*** | C→T | 0.87, 0.13 | Angiogenesis |
| *EDN1* | Endothelin 1 | rs5370  ***EDN*** | G→T | 0.82, 0.18 | Vasoconstriction |
| *IL-10* | Interleukin 10 | rs1800896  ***IL10-*** | G→A | 0.53, 0.47 | Immune Modulators/Cytokines |
| *HCFXI*  *(KLKB1)* | Human Coagulation Factor XI,  kallikrein B, plasma (Fletcher factor) 1 | rs925453  ***HCFX-*** | C→T | 0.67, 0.33 | Inflammatory response |
| *IGFR1* | Insulin-like growth factor 1 receptor | rs2016347  ***IGFR1-*** | T→G | 0.55, 0.45 | Cell Growth and Development |
